# Primary hyperparathyroidism presenting as a brown tumor in the mandible: a case report

**DOI:** 10.1186/s12902-019-0480-2

**Published:** 2020-01-13

**Authors:** Bojin Xu, Jie Yu, Yingli Lu, Bing Han

**Affiliations:** 1 Institute and Department of Endocrinology and Metabolism, Shanghai Ninth People’s Hospital Affiliated to Shanghai Jiao Tong University School of Medicine, Shanghai, China; 2 Department of Endocrinology, Shanghai Tongren Hospital, Affiliated to Shanghai Jiao Tong University School of Medicine, Shanghai, China

**Keywords:** Brown tumor, Primary hyperparathyroidism, Mandible, Osteolytic lesions, Case report

## Abstract

**Background:**

Primary hyperparathyroidism is characterized by hypercalcemia and elevated or inappropriately normal serum levels of parathyroid hormone. Brown tumor of bone is a rare non-neoplastic lesion resulted from abnormal bone metabolism in hyperparathyroidism. However, nowadays, skeletal disease caused by primary hyperparathyroidism is uncommon. We report a case of brown tumor in the mandible as the initial exhibition of primary hyperparathyroidism associated with an atypical parathyroid adenoma.

**Case presentation:**

The patient was a 49-year-old female, she had a pain mass on the right mandible a year ago and was treated with root canal therapy and marginal resection. After seven months, the mass recurred and enlarged. Enhanced CT scan, laboratory examination, Ultrasonography, ^99m^Tc-MIBI SPECT-CT scintiscan and pathological examination were used to confirm the diagnosis of brown tumor. The patient’s symptom improved after parathyroidectomy.

**Conclusions:**

^99m^Tc-MIBI SPECT/CT scintigraphy is a highly sensitive examination of the localization diagnosis of hyperparathyroidism. Brown tumors should be considered in the differential diagnosis of osteolytic lesions to avoid unnecessary and harmful interventions.

## Background

Primary hyperparathyroidism (PHPT) is a common endocrine disorder that is characterized by hypercalcemia and elevated or inappropriately normal serum levels of parathyroid hormone [1]. Single benign parathyroid adenoma is the most common cause of this disease (about 80% of the patients), whereas four gland parathyroid hyperplasia accounts for approximately 15–20% [2], multiple parathyroid adenomas for 5% and parathyroid cancer for < 1% of cases [1]. Brown tumor of bone, a rare non-neoplastic lesion resulted from abnormal bone metabolism in hyperparathyroidism, mostly affects facial bones, clavicle, ribs, pelvis, and femur [3]. When brown tumors associate with PHPT, they are most frequently caused by adenomas [4]. However, skeletal disease secondary to PHPT is rare nowadays [5].

We report a case of brown tumor in the mandible as the initial exhibition of PHPT associated with an atypical parathyroid adenoma.

## Case presentation

A 49-year-old female was referred to our hospital for a recurrent right mandible mass one year after primary excision. One year ago, she had a painful mass on the right mandible, which was first treated with root canal therapy and then by marginal resection of the right mandible at another medical institution. The postoperative pathology suggested giant cell granuloma. Seven months after surgery, the mass recurred and gradually enlarged.

**Fig. 1:**
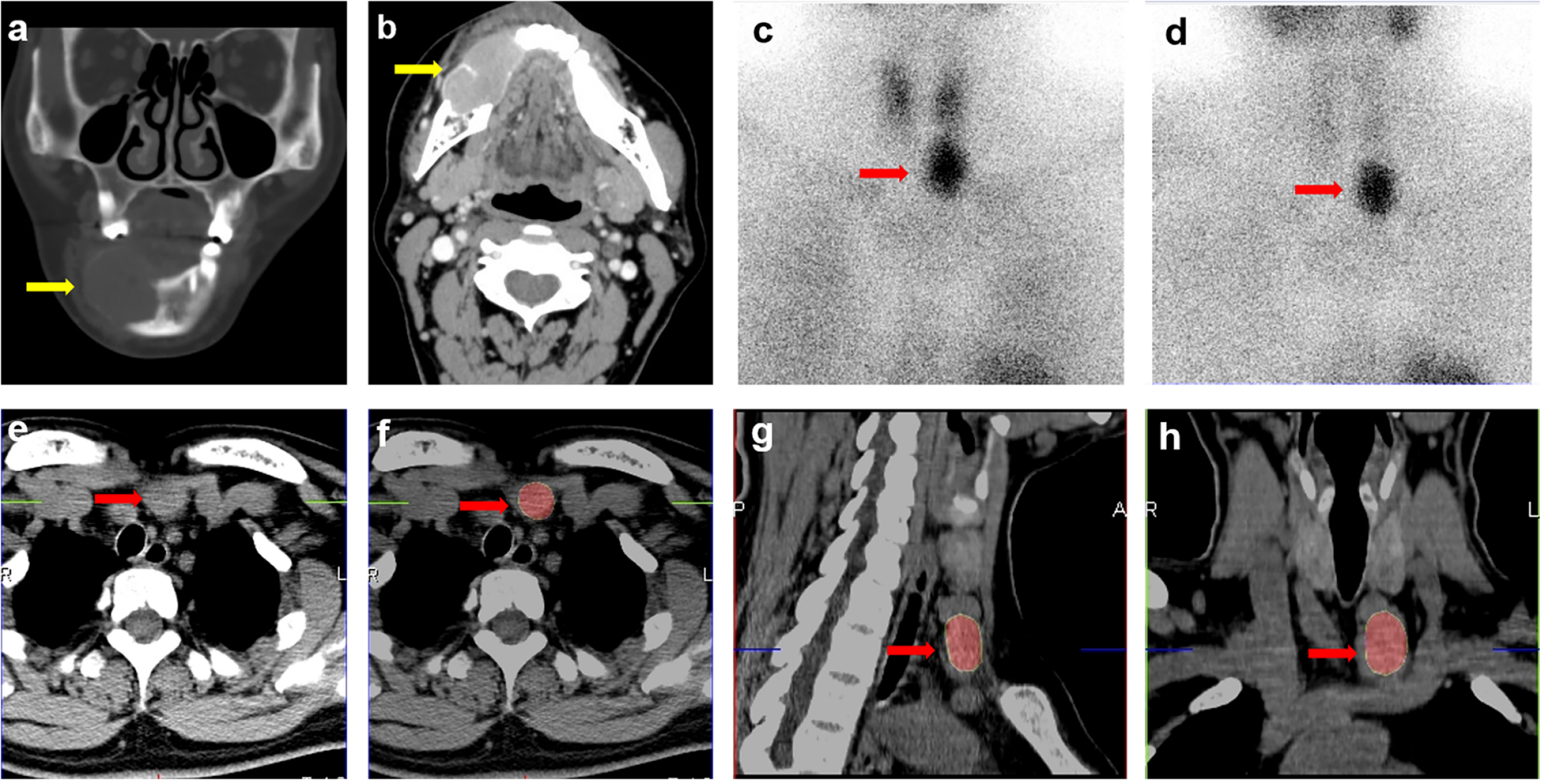
(**a**-**b**) Enhanced computer tomography scan of the brown tumor in the mandible (yellow arrows). (**a**) Coronal CT and (**b**) axial CT. (**c**-**d**) ^99m^Tc-MIBI of the parathyroid adenoma (red arrows): the early imaging (**c**) shows the radioactive concentration at the site of the left parathyroid, and the delayed imaging (**d**) shows that it did not degrade. (**e**-**h**) SPECT/CT scintigraphy revealed the low-density lesion with high radioactive uptake at the site of the left parathyroid (red arrows): (e-f) Axial, (**g**) sagittal, and (**h**) coronal images of the parathyroid adenoma

On admission, an enhanced CT scan of the patient revealed a 3.6*2.1 cm lesion on the right mandible (Fig. 1a, b). A review of her previous pathological section revealed that the right mandible was rich in osteoclast-like polykaryotic giant cells. Based on this observation, the patient was further evaluated. Laboratory examination showed hypercalcemia: 2.81 mmol/l (normal range: 2.08–2.71 mmol/l), hypophosphatemia: 0.66 mmol/l (normal range: 0.81–1.45 mmol/l), and a high PTH level: 916.0 pg/ml (normal range: 11.0–67.0 pg/ml). Ultrasonography revealed a hypoechoic mass on the left inferior thyroid lobe. ^99m^Tc-MIBI SPECT-CT scintiscan demonstrated increased radiotracer uptake at the site of the left parathyroid, which was suggestive of parathyroid adenoma (Fig. 1c-h). Then, the patient underwent left parathyroidectomy surgery, and the pathological findings confirmed the diagnosis of parathyroid adenoma. Postoperative follow-up showed normal serum calcium and PTH levels, and the mandible mass decreased gradually.

## Discussion and conclusions

In this case, the combination of hyperparathyroidism and the existence of giant cells in the mandibular pathology as well as gradual degradation of the tumor after operation supported the diagnosis of brown tumor.

Hyperparathyroidism may be primary, secondary and tertiary. Primary hyperparathyroidism is characterized by excessive PTH production and hypercalcemia, most frequently due to parathyroid adenoma. Secondary hyperparathyroidism is usually caused by vitamin D deficiency, malabsorption, or hypercalciuria. Low serum calcium levels resulted from primary diseases brings about redundant secretion of PTH. Tertiary hyperparathyroidism, in most cases, developed from secondary hyperparathyroidism and evolves into a more severe circumstance with autonomous PTH secretion [1]. As in our case, laboratory findings showed hypercalcemia, hypophosphatemia and a high PTH level, which were consistent with PHPT.

Nowadays, with the increasingly available measurement of biochemical examination, the detection rate of hyperparathyroidism has been increased and PHPT has evolved into a typically asymptomatic disease, especially in developed countries where serum calcium levels are routinely measured [1, 2, 6]. A more extensive serum calcium screening in the general population may help to identify and treat the patients at an early stage.

Brown tumors are giant cell lesions caused by abnormal bone metabolism in hyperparathyroidism. Increased circulating PTH aggravates osteoclastic bone absorption, then leads to diffuse osteopenia, fractures or multiple circumscribed lytic lesions [7, 8]. There is dark, reddish-brown coloration induced by prominent intralesional hemorrhage and hemosiderin deposition, thus gives the lesion its name [9]. These bone-resorbing lesions can occur in any part in the bone, but they are rarely the initial signs of hyperparathyroidism [10]. They are believed to be gradually dissolved after surgical resection of the parathyroid gland [11].

The most prominent aspect of this case was the discovery of a brown tumor in the mandible, which was the initial exhibition of an atypical parathyroid adenoma. However, the confirmed diagnosis was made after the surgery in the pathological findings, how to obtain certain diagnosis before the parathyroidectomy surgery remains to be explored. ^99m^Tc-MIBI SPECT/CT scintigraphy is a highly sensitive examination of the localization diagnosis of hyperparathyroidism [12]. We suggest that clinicians bear in mind that brown tumors should be considered in the differential diagnosis of osteolytic lesions to avoid unnecessary and harmful interventions.

## Data Availability

The datasets analyzed during the current study are available from the corresponding author on reasonable request.

## Abbreviations

^99m^Tc-MIBI: ^99m^Tc-sestamibi
CT: computed tomography
PHPT: Primary hyperparathyroidism
PTH: parathyroid hormone
SPECT: Single-Photon Emission Computed Tomography
